# Conversion of a *recA*-Mediated Non-toxigenic *Vibrio cholerae* O1 Strain to a Toxigenic Strain Using Chitin-Induced Transformation

**DOI:** 10.3389/fmicb.2019.02562

**Published:** 2019-11-07

**Authors:** Shrestha Sinha-Ray, Meer T. Alam, Satyabrata Bag, J. Glenn Morris Jr., Afsar Ali

**Affiliations:** ^1^Emerging Pathogens Institute, University of Florida, Gainesville, FL, United States; ^2^Department of Microbiology and Cell Science, College of Agricultural and Life Sciences, University of Florida, Gainesville, FL, United States; ^3^Department of Environmental and Global Health, College of Public Health and Health Professions, University of Florida, Gainesville, FL, United States; ^4^Department of Medicine, School of Medicine, University of Florida, Gainesville, FL, United States

**Keywords:** *Vibrio cholerae*, chitin-induced natural transformation, RS1-CTX-TLC prophages acquisition, cholera toxin, Haiti, aquatic reservoirs

## Abstract

Toxigenic *Vibrio cholerae* strains, including strains in serogroups O1 and O139 associated with the clinical disease cholera, are ubiquitous in aquatic reservoirs, including fresh, estuarine, and marine environments. Humans acquire cholera by consuming water and/or food contaminated with the microorganism. The genome of toxigenic *V. cholerae* harbors a cholera-toxin producing prophage (CT-prophage) encoding genes that promote expression of cholera toxin. The CT-prophage in *V. cholerae* is flanked by two satellite prophages, RS1 and TLC. Using cell surface appendages (TCP and/or MSHA pili), *V. cholerae* can sequentially acquire TLC, RS1, and CTX phages by transduction; the genome of each of these phages ultimately integrates into *V. cholerae’s* genome in a site-specific manner. Here, we showed that a non-toxigenic *V. cholerae* O1 biotype El Tor strain, lacking the entire RS1-CTX-TLC prophage complex (designated as RCT: R for RS1, C for CTX and T for TLC prophage, respectively), was able to acquire RCT from donor genomic DNA (gDNA) of a wild-type *V. cholerae* strain (E7946) via chitin-induced transformation. Moreover, we demonstrated that a chitin-induced transformant (designated as AAS111) harboring RCT was capable of producing cholera toxin. We also showed that *recA*, rather than *xerC* and *xerD* recombinases, promoted the acquisition of RCT from donor gDNA by the recipient non-toxigenic *V. cholerae* strain. Our data document the existence of an alternative pathway by which a non-toxigenic *V. cholerae* O1 strain can transform to a toxigenic strain by using chitin induction. As chitin is an abundant natural carbon source in aquatic reservoirs where *V. cholerae* is present, chitin-induced transformation may be an important driver in the emergence of new toxigenic *V. cholerae* strains.

## Introduction

Cholera, an ancient diarrheal disease characterized by profuse secretory diarrhea and caused by toxigenic strains of *Vibrio cholerae*, is a major public health concern in developing and poor countries lacking safe drinking water, sanitation and hygiene (Barua, 1992; Kaper et al., 1995; Faruque et al., 1998). Moreover, the disease is often associated with poverty, and internal and external displacement of people due to wars and natural disasters (Morris, 2011; Baker-Austin et al., 2018). A comprehensive study estimated that cholera causes between 1.3 and 4 million cases with 120,000 deaths globally per year, with major recent “hot spots” in Africa, Asia and Hispaniola (Ali et al., 2012). Of 220 known and confirmed serogroups of *V. cholerae*, clinical cholera is associated almost exclusively with strains in serogroups O1 and O139 (Albert, 1994; Nair et al., 1994; Kaper et al., 1995).

Toxigenic *V. cholerae* carries two key genetic elements: cholera toxin genes (*ctxAB*) and a toxin co-regulated pilus gene (*tcpA*); both of these virulence elements are under the control of a master regulator, ToxR (Miller et al., 1987; Herrington et al., 1988; Peterson and Mekalanos, 1988). Cholera toxin (CT) is encoded by cholera toxin genes that are harbored by a filamentous phage (CTXϕ) integrated as a CTX prophage into the genome of toxigenic strains of *V. cholerae* (Waldor and Mekalanos, 1996). The *tcpA* gene encoding TcpA protein is a component of Vibrio Pathogenicity Island I (VPI-I); TcpA is required for the colonization of *V. cholerae* by human intestinal epithelial cells (Thelin and Taylor, 1996; Karaolis et al., 1998). In a canonical toxigenic *V. cholerae* El Tor strain, CTX prophage is flanked by RS1 (left) and TLC (right) prophages. RS1 and TLC prophages encode the genome of RS1ϕ and TLCϕ, respectively (Davis et al., 2002; Hassan et al., 2010; Das, 2014). In addition to being chromosomally integrated prophages, RS1, CTX, and TLC can form a replicative form (RF) (Waldor and Mekalanos, 1996; Faruque et al., 2002; Hassan et al., 2010). While CTXϕ exploits *V. cholerae*’s TcpA pilus as receptor, TLCϕ and RS1ϕ use the bacterium’s MSHA pilus and MSHA/TcpA pilus as receptor, respectively (Waldor and Mekalanos, 1996; Faruque and Mekalanos, 2012; Das, 2014). Once the genomes of these phages are released into *V. cholerae’s* cells, the phage genomes integrate into the chromosome of *V. cholerae* in site-specific manner with TLCϕ being the first followed by RSIϕ and CTXϕ genomes (Hassan et al., 2010; Faruque and Mekalanos, 2012). A component of *V. cholerae* genome harboring both a defective *dif1* site and XerC and XerD recombinase-binding sites allows the sequential integration of these phage genomes. Interestingly, *dif1* site required for the successful dimer resolution of *V. cholerae* chromosome, is flanked by sequences that serve as binding sites for XerC and XerD recombinases (McLeod and Waldor, 2004; Faruque and Mekalanos, 2012).

Chitin, a naturally occurring complex biopolymer and the second most abundant carbon source in nature, is frequently found to be associated with exoskeletons of shellfish and crustaceans and provides a nutrient source for *V. cholerae* (Colwell and Huq, 1994; Meibom et al., 2004; Elieh-Ali-Komi and Hamblin, 2016). Chitin also promotes natural competency for *V. cholerae*, facilitating horizontal gene transfer (Meibom et al., 2005). In an experiment mimicking aquatic reservoirs, a typical *V. cholerae* O1 El Tor strain acquired the entire *V. cholerae* O139 O-antigen-encoding genetic region by using chitin induction (Blokesch and Schoolnik, 2007). Furthermore, a toxigenic *V. cholerae* El Tor strain carrying CTX^ET^ prophage was converted to a hybrid toxigenic strain [El Tor biotype strain carrying classical CTX prophage, CTX^class^] by using chitin induction (Udden et al., 2008). However, the role of chitin in the transformation of non-toxigenic *V. cholerae* O1 strains lacking the entire RSI, CTX, and TLC prophage complex (RCT) to a fully toxigenic O1 strain remains unknown. Here we show that chitin induction promoted the transfer of the entire RCT from genetically marked (kan^R^) donor genomic DNA (gDNA) to a non-toxigenic *V. cholerae* strain, rendering the recipient strain toxigenic. We also demonstrated that RecA, not XerC and XerD recombinases, facilitated this toxigenic conversion.

## Materials and Methods

### Bacterial Strains, Plasmids, and Growth Conditions

Bacterial strains and plasmids used in this study are listed in Table 1. As needed, *V. cholerae* and *Escherichia coli* strains of interest were subcultured from glycerol broth stored at −80°C to Luria-agar (L- agar) and the cultures were incubated statically overnight at 37°C incubator. Unless otherwise indicated, for growth in Luria-broth (L- broth), a single colony of microorganism grown overnight on L-agar was transferred into L-broth and the culture was grown overnight at 37°C with a shaking speed of 250 rpm in an orbital shaker. As required, antibiotics were used at the following concentrations: rifampicin (50 μg/ml), kanamycin (50 μg/ml), spectinomycin (100 μg/ml) and ampicillin (100 μg/ml). For protein expression assay, the *recA* gene was cloned into pBAD/His A plasmid (Thermo Fischer Scientific, Waltham, MA, United States) and the expression of RecA recombinant protein was induced in culture by adding arabinose at a final concentration of 0.2%.

**TABLE 1:** Bacterial strains and plasmids used in this study.

**Strain/Plasmid**	**Description/genotype**	**References**
***V. cholerae***		
E7946	O1 El Tor, ogawa clinical toxigenic isolate in 1978 from Bahrain	Laboratory collection
N16961	O1 El Tor, inaba clinical toxigenic isolate in 1971 from Bangladesh	Heidelberg et al., 2000
O395	O1 classical, ogawa clinical toxigenic isolate in 1965 from India	Laboratory collection
HC16	O1 El Tor Ogawa clinical toxigenic isolate in 2012 from Haiti	Rahman et al., 2014
HC1037	O1 El Tor Ogawa clinical toxigenic isolate in 2014 from Haiti	Duncan et al., 2018
Env-2	Non-O1/Non-O139 environmental non-toxigenic isolate in 2012 from Haiti	Rahman et al., 2014
Env-9	O1 Ogawa environmental non-toxigenic isolate in 2012 from Haiti	Azarian et al., 2016
AAS35	Rifampicin resistant version of Env-9 created by spontaneous mutation, rif^R^	This study
AAS56	E7946 Δ *orfU*::*kan*^*R*^	This study
AAS69	AAS35 Δ *xerC*::*kan*^*R*^, rif^R^	This study
AAS70	AAS35 Δ *xerD*::*kan*^*R*^, rif^R^	This study
AAS72	E7946 Δ *orfU*::*spec*^*R*^	This study
AAS74b	AAS35 Δ *recA*::*kan*^*R*^, rif^R^	This study
AAS91	AAS35::*RS1-CTX-TLC*Δ *orfU*::*kan*^*R*^, rif^R^	This study
AAS93	AAS35::*RS1-CTX-TLC*Δ *orfU*::*spec*^*R*^, rif^R^	This study
AAS111	AAS35::*RS1-CTX-TLC*Δ *xerC*::*kan*^*R*^, rif^R^	This study
AAS125	E7946 Δ *xerC*::*kan*^*R*^	This study
***E. coli***		
DH5α	*recA* Δ *lac*U169 ϕ80d *lacZ*Δ M15	Gibco, BRL
**Plasmids**		
pBAD/His A	pBR322 origin, expression vector, amp^R^	Thermo fisher scientific
pSMA4	1065 bp *recA* ORF of E7946 was cloned into *Xho*I-*Eco*RI sites of pBAD/His A, amp^R^	This study

### Splicing by Overlap Extension (SOE) PCR

We used splicing by overlap extension (SOE) PCR and multiplex genome editing by natural transformation to create our desired mutation(s) in *V. cholerae* strains as described previously (Marvig and Blokesch, 2010; Dalia et al., 2014). For example, to create a null mutation in the *orfU* gene (1284-bp) in CTX prophage in the background of *V. cholerae* O1 strain E7946 (Table 1), a two-step SOE PCR amplification protocol was engineered. The first step PCR cycle involved in the amplification of 1,303-bp kan^R^ cassette (Dalia et al., 2014) as well as the upstream (2,754-bp) and downstream (2,659-bp) fragments of *orfU* gene using three sets of convergent PCR primers (aali 563/aali 564, aali 651/aali 655 and aali 656/aali 654, respectively). The inner set of primers amplifying the upstream (aali 655) and downstream (aali 656) regions of homology, were designed in such a way that the 5′ ends of each of that primers overlapped the primers amplifying the kanamycin cassette kan^R^ (aali 563 and aali 564). The upstream and downstream fragments of *orfU* gene were amplified from gDNA of E7946 strain; gDNA of E7946 was extracted and purified using High Pure PCR Template Preparation Kit (Roche Life Science, Indianapolis, IN, United States). All the PCR primers are listed in Supplementary Table S1. Q5 High-Fidelity DNA Polymerase (New England Biolabs Inc., Ipswich, MA, United States) was used to amplify the desired DNA fragments and the PCR conditions were as follows: initial denaturation at 98°C for 3 min, 30 cycles each of denaturation at 98°C for 30 sec, annealing at 60°C for 45 sec and elongation at 72°C for 2 min, and final extension at 72°C for 5 min followed by hold at 4°C. In the second step PCR cycle, three PCR fragments obtained from first PCR cycle described above were fused together using Phusion High Fidelity DNA Polymerase (Thermo Fischer Scientific, Waltham, MA, United States) that yielded a large 6,718-bp PCR product encompassing OrfUF1- kan^R^ -OrfUF2. The PCR conditions for this step were as follows: initial denaturation at 98°C for 2 min, 25 cycles each of denaturation at 98°C for 10 s, annealing at 66°C for 30 s and elongation at 72°C for 3.5 min, and final extension at 72°C for 5 min followed by hold at 4°C.

### Chitin-Induced Transformation of *V. cholerae* Using SOE PCR Product

The 6,718-bp PCR product obtained by SOE PCR described above was gel purified using QIAquick Gel Extraction Kit (Qiagen, Hilden, Germany) and the DNA was used to knock out the *orfU* gene from wild-type *V. cholerae* E7946 strain by using chitin-induced transformation as described previously (Meibom et al., 2005; Marvig and Blokesch, 2010) with minor modifications. Briefly, a single colony of recipient wild-type *V. cholerae* strain (E7946) grown overnight on L-agar at 37°C was transferred into 3 ml L-broth, and the culture was incubated overnight at 30°C with a shaking speed of 250 rpm. Next day, the culture was diluted in fresh L-broth (1:100 [v/v]) and grown at 30°C until it reached a value between 0.4 and 0.5 at OD_600_. One ml of the culture was harvested, washed, and finally resuspended in 0.1 ml of filter-sterilized instant ocean water (IOW) (7 g/liter). Then the resuspended culture was added to 50–60 mg of sterilized (autoclaved) chitin (Sigma-Aldrich, St. Louis, MO, United States) along with 0.9 ml fresh filter sterilized IOW in a 1.5 ml micro-centrifuge tube; the culture was mixed thoroughly and incubated statically at 30°C for 16–24 h. Following incubation, ∼400–500 μl of spent IOW was removed carefully followed by the addition of fresh ∼400–500 μl IOW. Two hundred ng of PCR product (OrfUF1-kan^R^-OrfUF2) was added as the donor DNA to the culture followed by gentle mixing. The culture was further incubated statically overnight at 30°C; the cells were detached from chitin by vigorous vortexing and 0.5 ml was transferred to 1 ml fresh L-broth and the culture was incubated at 37°C for 2 h. Following incubation, the culture was plated on L-agar supplemented with kanamycin and the plates were incubated overnight at 37°C. The potential kanamycin resistant transformants designated as AAS56 (E7946 Δ *orfU*::*kan*^*R*^) (Table 1) were screened and confirmed by PCR and DNA sequencing. Transformation frequency was determined by counting the number of colony-forming units (CFUs) grown on plates supplemented with antibiotic(s) by dividing the number of total CFUs obtained from plates with no added antibiotic. Other mutations, including, AAS69 (Env-9 rif^R^ Δ *xerC*::*kan*^*R*^), AAS70 (Env-9 rif^R^ Δ *xerD*::*kan*^*R*^) and AAS74b (Env-9 rif^R^ Δ *recA*::*kan*^*R*^) (Table 1) null mutants were created in the background of AAS35 (Env-9 rif^R^) by inserting a kan^R^ resistance cassette using SOE PCR as described above. For experimental convenience, AAS72 (E7946 Δ *orfU*::*spec*^*R*^) (Table 1) was also created by introducing a spectinomycin resistance marker replacing the *orfU* gene. All primers used to create SOE PCR constructs are listed in Supplementary Table S1.

### Chitin-Induced Transformation of *V. cholerae* Using Genomic DNA (gDNA)

In addition to SOE PCR product(s) described above, genomic DNA (gDNA) was also used to transform *V. cholerae* strains using chitin-induced transformation. For example, 2 μg of the donor (AAS56) gDNA was added to the culture of AAS35 lacking the entire RCT, in presence of chitin as described above. Experimental conditions involving chitin-induced transformation were similar to what has been described above concerning the SOE PCR product(s). The potential kan^R^ transformant designated as AAS91 (Table 1) was selected on L-agar supplemented with kanamycin. To detect if the entire RCT had incorporated between *rtxA* gene and VC1479 in the transformant, fourteen sets of convergent PCR primers were designed to confirm the incorporation of all genes encoding RCT prophages. The gDNA of AAS91 was also used to transform a fresh AAS35 using chitin-induced transformation. As control, we also used two clinical Haitian strains (HC16 and HC1037) in transformation experiments (Tables 1, 2); both HC16 and HC1037 carry an integrating conjugative element (ICE)-encoded DNase gene called *ideA* that limits the chitin-induced transformation in circulating toxigenic Haitian *V. cholerae* O1 strains using exogenous gDNA (Dalia et al., 2015). Furthermore, to determine if XerC, XerD, and RecA were required for the uptake of RCT during chitin-induced transformation, the gDNA of AAS72 (Table 1) was used to assess whether AAS69, AAS70, and AAS74b (Table 1) can independently acquire RCT via chitin-induced transformation. AAS35 was used as a positive control for RCT acquisition during chitin-induced transformation.

**TABLE 2:** Transformation of *Vibrio cholerae* Env-9 rif^R^ (AAS35) strain with genomic DNA (gDNA) of donor *V. cholerae* strains with chitin induction.

**Donor gDNA**	**Recipient strain**	**Range of transformation frequency^a^**
AAS56 [E7946 Δ *orfU*::*kan*^*R*^]	Env-9 rif^R^	9.72 × 10^–6^ to 3.08 × 10^–7^
None	Env-9 rif^R^	0
AAS56 [E7946 Δ *orfU*::*kan*^*R*^]	None	0
AAS91[Env-9 rif^R^ Δ *orfU*::*kan*^*R*^]	Env-9 rif^R^	7.72 × 10^–6^ to 1.56 × 10^–7^
AAS56 [E7946 Δ *orfU*::*kan*^*R*^]	HC16	3.02 × 10^–8^ to 2.55 × 10^–9^
AAS56 [E7946 Δ *orfU*::*kan*^*R*^]	HC1037	1.05 × 10^–8^ to 1.53 × 10^–9^

*^a^Data represent multiple independent transformation experiments.*

### Co-transformation of Env-9 rif^R^ Δ *orfU*::*spec*^*R*^ With Wild-Type *orfU* Gene for Replacement of the Spectinomycin Cassette

The strain AAS93 (Env-9 rif^R^ RCT Δ *orfU*::*spec*^*R*^) was created using the gDNA of AAS72 (Table 1) and chitin-induced transformation; in subsequent studies, the *spec*^*R*^ marker of AAS93 was replaced with the wild-type *orfU* gene using co-transformation and chitin-induced transformation as described previously (Dalia et al., 2014). Briefly, this requires two unlinked markers: while one marker was used for selection purpose, the other one was used for the screening of the integration of an unselected marker. For our experimental purpose, a 6892-bp PCR product (XerCF1-kan^R^-XerCF2) constructed by SOE strategy was used to create a null mutation of *xerC* with the insertion of a kanamycin resistance marker (selected). The second 6,718-bp PCR product (OrfUF1-*orfU*-OrfUF2) containing the wild-type *orfU* gene and upstream and downstream arms of homology (unselected) was used to replace the *spec*^*R*^ marker of AAS93. The AAS93 was grown in the presence of chitin and IOW for 20 h. The next day, approximately 3 μg of the unselected PCR product (OrfUF1-*orfU*-OrfUF2) was added followed by 30 ng of the selected PCR product (XerCF1-kan^R^-XerCF2). After 16–20 h of incubation at 30°C, the cells were detached from the chitin surface by vigorously vortexing for 30 s. Transformants were selected on L-agar plate supplemented with rifampicin and kanamycin. The resulting transformant designated as AAS111 (Env-9 rif^R^ RCT Δ *xerC*::*kan*^*R*^) (Table 1) was verified by spectinomycin sensitivity and PCR for the presence of wild-type *orfU* gene and *xerC* null mutation followed by DNA sequencing.

### Gene Cloning, Transformation, and Electroporation

To clone the *recA* gene in pBAD/His A vector, restriction endonuclease sites, including *Xho*I or *Eco*RI were introduced at the 5′ end of two convergent PCR primers, designated as aali 1053 and aali 1054, respectively (Supplementary Table S1). The *recA* gene consisting of 1,065-bp was PCR amplified using these PCR primers, *V. cholerae* E7946 genomic DNA as template, and Q5 High-Fidelity DNA Polymerase. The resulting PCR amplicon was gel purified with a QIAquick gel extraction kit, digested with *Xho*I and *Eco*RI, and ligated with similarly digested pBAD/His A vector (Table 1). The ligated product was then transformed into *E. coli* DH5α cells (Table 1) that were made competent using calcium chloride treatment as described previously (Jubair et al., 2014). The transformant resulting from cloning experiment was designated as pSMA4 (Table 1), and the transformant was confirmed to determine if it has cloned desired gene by restriction enzyme digestion and DNA sequencing.

For the complementation assay, the plasmid (pSMA4) was introduced into the *V. cholerae recA* mutant [AAS74b]; (Table 1) using electroporation (Pant et al., 2016) with minor modifications. Briefly, the *V. cholerae recA* mutant was made electro-competent following harvesting, washing and resuspending the culture in filter sterilized G buffer (137 mM sucrose, 1 mM HEPES, pH 8.0). Plasmid DNA (700-1000 ng) obtained from pSMA4 was mixed with the competent *V. cholerae* cells in a microcentrifuge tube (pre-chilled), and the mixture was immediately transferred into a pre-chilled 0.2 cm electroporation cuvette. The electroporation was performed with an electrical pulse of 2000 volt for five milliseconds in a Gene Pulser Xcell^TM^ Electroporation System (Bio-Rad, United States). Following electric pulse, *V. cholerae* cells were immediately grown in L-broth at 37°C with a shaking speed of 250 rpm for 1 h; the culture was then plated onto L-agar plate supplemented with ampicillin. Transformants were confirmed by plasmid extraction followed by restriction analysis as describe previously (Ali et al., 2000).

### Whole Genome Sequencing, Assembly, and Genomic Comparison

PacBio Sequel V3 chemistry was used for the whole genome sequencing of *V. cholerae* AAS91 as per manufacturer’s instructions. Briefly, purified genomic DNA of AAS91 was sheared using Covaris G-tube to get around 30 kb fragments that were used for the SMRTbell library preparation: Exonuclease VII treatment, DNA Damage Repair, DNA end repair, Blunt-end ligation of SMRTbell adaptors, and Exonucleases III/VII digestion. The library was loaded onto the PacBio Sequel sample plate for sequencing.

The raw reads generated from the PacBio Sequel sequencing platform were analyzed with PacBio SMRT Analysis software (6.0.0.47836). The demultiplexed reads with a length >100 bp were processed by HGAP4 (Hierarchical genome assembly process) and Canu (Koren et al., 2017) with optimized parameters to generate *de novo* genome chromosomes. Both assemblers automatically filter the sequencing data to remove SMRTbell adapter sequences and recover high-quality genomic content. The initial assemblies were circularized by using the Circulator tool (Hunt et al., 2015). Once circularized, the assemblies were imported into SMRT Link for subsequent polishing with the Resequencing Analysis to attain a higher base quality. The samtools, fastx-toolkit, and R-based scripts developed at ICBR, University of Florida were used to compare the assembled chromosomes with the reference genome of Env-9 (NCBI: NZ_CP012997.1 and NZ_CP012998.1). The identification and analysis of similarities and differences arising from comparisons of genomes were plotted by using Circos (Krzywinski et al., 2009). The annotated whole genome sequences for AAS91 has been deposited in GenBank (accession nos. CP042299 and CP042300).

### Cholera Toxin (CT) Assay

For *in vitro* assessment of cholera toxin (CT) produced by *V. cholerae* strains, the microorganisms were grown in AKI medium (1.5% Bacto peptone, 0.4% yeast extract, 0.5% NaCl and 0.3% sodium bicarbonate) as described previously (Iwanaga et al., 1986). Briefly, a single colony of *V. cholerae* strain of interest grown overnight on L-agar at 37°C was inoculated in 10 ml AKI medium in a culture tube and the culture was grown statically at 37°C for 4 h. Then all 10 ml culture was transferred aseptically to a sterile conical flask (100 ml) and the culture flask was incubated at 37°C with a shaking speed of 250 rpm for 16 h. The culture was harvested by centrifugation at 14,000 × *g* for 20 min at 4°C and the supernatant containing CT was aseptically removed to a sterile centrifuge tube. As described previously (Almeida et al., 1990), CT was measured by GM1 - enzyme linked immunosorbent assay (ELISA) using pure CT and phosphate-buffered saline (PBS) as positive and negative controls, respectively. 2 μg/ml GM1 ganglioside in PBS was used for coating the microtiter wells in a 96-well microtiter plate. Antibody to CT raised in goat was diluted to 1:2000-fold; alkaline phosphatase-labeled anti-goat conjugate was diluted to 1:1000-fold in PBS containing 0.1% BSA. The preparations were used in this assay. *p*-nitrophenyl phosphate was used as the substrate and OD_405_ was measured in a plate reader (SynergyMx, BioTek, United States). A standard curve (SC) of known CT concentrations was plotted and the SC was used to estimate the amount of CT present in each sample. Total ng of CT produced per ml of culture per OD_600_ unit (ng ml^–1^ OD_600_^–1^) was determined for biological triplicates with three technical replicates for each strain and the figure was drawn using GraphPad Prism 8 software.

### Statistical Analysis

All statistical analyses were performed with GraphPad Prism 8 software. Data were expressed as means with standard deviation and analyzed using one-way analysis of variance (ANOVA) with Tukey’s multiple comparison test. *p* < 0.05 was considered statistically significant.

## Results

### Chitin-Induced Transformation Promoted the Transfer of RS1-CTX-TLC (RCT) Prophages to a Non-toxigenic *V. cholerae* O1 Strain

**FIGURE 1:**
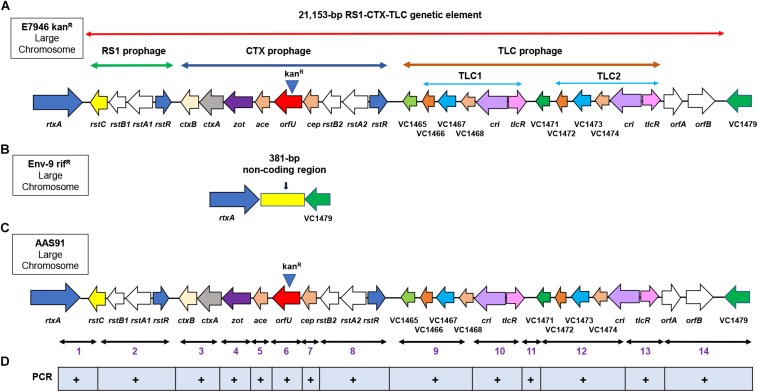
Genetic arrangement of RS1, CTX and TLC (RCT) prophages in the large chromosome of *Vibrio cholerae* O1 strain. **(A)** genomic DNA (gDNA) of a wild-type *V. cholerae* E7946 strain was used as the donor DNA in chitin-induced transformation, encompassing 21,153-bp encoding RCT which was genetically marked with a kanamycin resistance cassette (kan^R^) (note, kan^R^ at the top of *orfU* gene) replacing *orfU* gene in CTX prophage. The arrows show either gene name, VC followed by a number or ORF designation. **(B)** Env-9 rif^R^, served as a recipient strain in chitin-induced transformation, lacking the RCT prophages while retaining a 381-bp non-coding region that is present between *rtxA* (VC1451) and VC1479 encoding a hypothetical protein. **(C)** following chitin-induced transformation, Env-9 rif^R^ acquired kan^R^-marked RCT prophages mirror-imaging RCT of E7946 kan^R^; the newly created transformant was designated as AAS91. **(D)** fourteen convergent PCR primer sets were used to amplify 14 PCR fragments (1–14) confirming all 27 genes present in the RCT prophages both in donor E7946 and transformant AAS91strains. A plus (+) sign indicates presence of the amplicon sequences spanning the targeted region in RCT prophages.

We first examined if chitin-induced transformation promotes the transfer of RCT from a donor gDNA to a recipient non-toxigenic *V. cholerae* O1 strain lacking the entire RCT. To test this idea, we created a donor *V. cholerae* strain in the background of a wild-type E7946 strain (an O1 El Tor biotype strain). Briefly, we inserted a kanamycin resistance (kan^R^) gene cassette replacing the entire *orfU* gene in the CTX prophage in E7946 strain yielding AAS56 (Table 1 and Figure 1A). As a recipient strain we chose Env-9, a non-toxigenic *V. cholerae* O1 El Tor strain isolated from the Haitian aquatic environment (Azarian et al., 2016). The Env-9 lost the entire RCT prophage (21,153-bp encompassing 27 ORFs [VC1452 (*rstC*) to VC1478] while retaining a 381-bp non-coding region between *rtxA* gene and VC1479 (Figure 1B). Interestingly, the 381-bp non-coding region consisted of a defective *dif1* site with XerC and XerD recombinase-binding sites (Figure 1B and Supplementary Figure S1). For experimental convenience, we spontaneously genetically marked the Env-9 strain with rifampicin, yielding AAS35 [genotype, Env-9 rif^R^] (Table 1 and Figure 1B). For chitin-induced transformation assay, we extracted and purified genomic DNA (gDNA) from donor AAS56 strain (Table 1), and 2 μg of the gDNA was added to the recipient strain (AAS35) grown in IOW supplemented with chitin. Appropriate controls, including donor (AAS56) gDNA minus recipient strain (AAS35), and recipient strain (AAS35) minus donor gDNA (Table 2) in otherwise identical experimental condition were included. We also used two Haitian clinical *V. cholerae* strains (HC16 and HC1037) (Tables 1, 2) carrying *ideA* gene as another set of control. Following incubation, we selected potential transformants on L-agar supplemented with kanamycin and rifampicin as described in the methods section. We detected a transformant designated as AAS91 (genotype, Env-9 rif^R^ RCT Δ *orfU*::*kan*^*R*^) (Table 1 and Figure 1C) from multiple independent experiments with a transformation frequency ranging from 9.72 × 10^–6^ to 3.08 × 10^–7^ (Table 2). As expected, controls, including donor (AAS56) gDNA minus recipient strain (AAS35), and recipient strain (AAS35) minus donor gDNA yielded no transformants (Table 2). Interestingly, we observed that both HC16 and HC1037 yielded kan^R^ transformants with ∼100-fold lower frequency relative to AAS35 strain during chitin-induced transformation (Table 2). Our result is consistent with a previous report demonstrating that circulating Haitian *V. cholerae* O1 strains harboring *ideA* gene limits chitin-induced transformation by horizontal gene transformation [HGT](Dalia et al., 2015). We then asked if gDNA of AAS91 could transform a fresh AAS35 recipient strain. To test this, we extracted and purified gDNA of AAS91 and mixed it with AAS35 in presence of chitin. We did obtain transformants at a frequency similar to that seen with the original chitin-induced transformation assay (Table 2).

**FIGURE 2:**
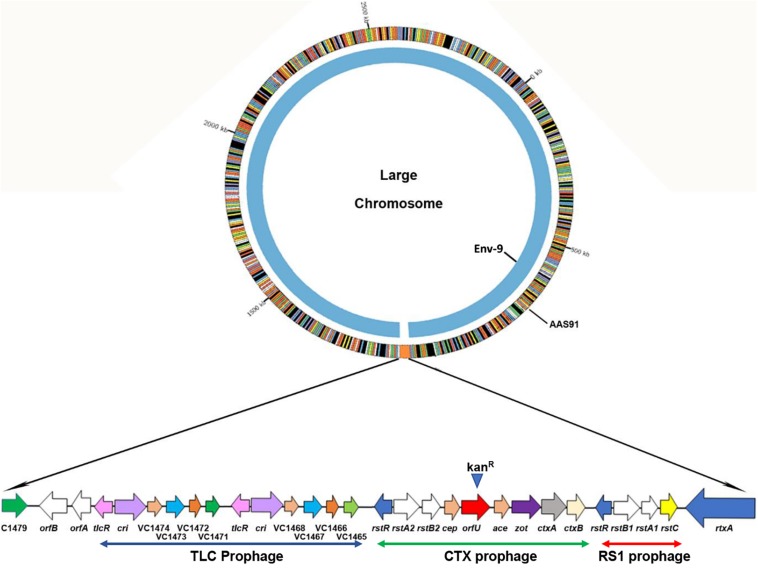
PacBio whole genome sequencing and sequence comparison of *Vibrio cholerae* AAS91 to that of its mother strain Env-9 (NCBI: NZ_CP012997.1 and NZ_CP012998.1) corroborated the successful integration of the 21,153-bp of RCT prophages between *rtxA* and VC1479 in AAS91 following chitin-induced transformation. We compared only the large chromosome where RCT is present. While Env-9 lacked RCT prophages (inner chromosome), AAS91 acquired entire RCT prophages as presented in extended form with arrows showing the genes comprising of RCT prophages.

To examine if all transformants acquired and integrated genomes of RCT prophages from donor strain, we randomly selected six transformants from different transformation experiments. Each transformant was subjected to PCR amplification targeting left (*rstC-rtxA*) and right (*orfA* to intergenic region between VC1479 and VC1478) junctions of RCT using convergent PCR primers, including aali 538/aali 537 and aali 528/aali 571, respectively. We purified and sequenced the PCR products; sequencing result confirmed that five (83%) of six transformants had successful integration of left and right junctions of potential RCT prophages between ORFs VC1451 and VC1479. To further ensure that all genes spanning entire RCT prophages had integrated between *rtxA* gene and VC1479, we designed fourteen sets of converging PCR primers (*n* = 28) that would confirm the presence of each gene encoded by RCT phages. Sequence analysis of each of 14 PCR products confirmed that the entire RCT was, indeed, incorporated between the *rtxA* gene and VC1479 (Figure 1D and Supplementary Figure S2). As expected, we also noted that the defective *dif1* site of AAS35 resolved into a functional *dif1* site in AAS91 (Supplementary Figure S1). The presence of the spacer region (band 11 in Supplementary Figure S2) and VC1471 (band 12 in Supplementary Figure S2) in the transformant confirmed the integration of two TLC prophages. To determine if all gene sequences belonging to RCT of donor (AAS56) strain remain unchanged in transformant (AAS91) following successful transformation, we performed whole genome sequencing [WGS] (PacBio sequencing) of the transformant (AAS91) with genome annotation. Bioinformatics analysis revealed that all gene sequences (base by base) encompassing donor RCT had been incorporated between the *rtxA* gene and VC1479 in AAS91 (Figure 2). Our data confirmed that chitin-induced transformation promotes the transfer of genetic elements encoding RCT prophages from a donor (AAS56) gDNA to a non-toxigenic *V. cholerae* O1 strain (AAS35), at a site adjacent to the *rtxA* gene rendering that non-toxigenic O1 strain to toxigenic O1 strain.

### RecA, Independent of XerC and XerD, Promoted the Integration of RCT Prophages Next to *rtxA* Gene

Previous reports (McLeod and Waldor, 2004; Faruque and Mekalanos, 2012) demonstrated that *V. cholerae*-encoded recombinases, including XerC and XerD, mediate the complex and sequential site specific integration of the genomes of TLCϕ, RS1ϕ, and CTXϕ, phages in the *V. cholerae* genome. To determine if *xerC* and *xerD* were also required for the integration of these prophages (RCT) at the *dif1* site of the recipient AAS35 strain during chitin-induced transformation, we created a null mutation in both the *xerC* and *xerD* genes in the wild-type AAS35 (recipient strain) as described in methods section. Interestingly, in contrast to previous reports (Faruque and Mekalanos, 2012), we observed that a mutation in the *xerC* and *xerD* genes (Supplementary Table S2) was unable to inhibit the integration of RCT prophages between *rtxA* and VC1479 in the AAS35 recipient strain during chitin-induced transformation. *recA* promotes canonical homologous recombination in many different bacterial species (Chen et al., 2008). To determine if *recA* promoted homologous recombination of the RCT prophages between *rtxA* and VC1479 in a wild-type recipient AAS35 strain, we created a null mutation in the *recA* gene in AAS35 strain yielding AAS74b. Strikingly, in contrast to *xerC* and *xerD* mutations, AAS74b strain completely inhibited chitin-induced transformation with donor gDNA obtained from AAS56 (E7946 kan^R^, Supplementary Table S2). Complementation of AAS74b mutant with a wild-type *recA* gene cloned and expressed in pBAD vector (pSMA4) restored transformation frequency when gDNA of AAS56 was used in chitin-induced transformation (Supplementary Table S2). Our data confirmed that RecA is required for the integration of the RCT prophages in recipient strains by homologous recombination during chitin-induced transformation.

### Transformation of Wild-Type Env-9 rif^R^ Strain With RCT Prophages Enabled the Strain to Produce Cholera Toxin

**FIGURE 3:**
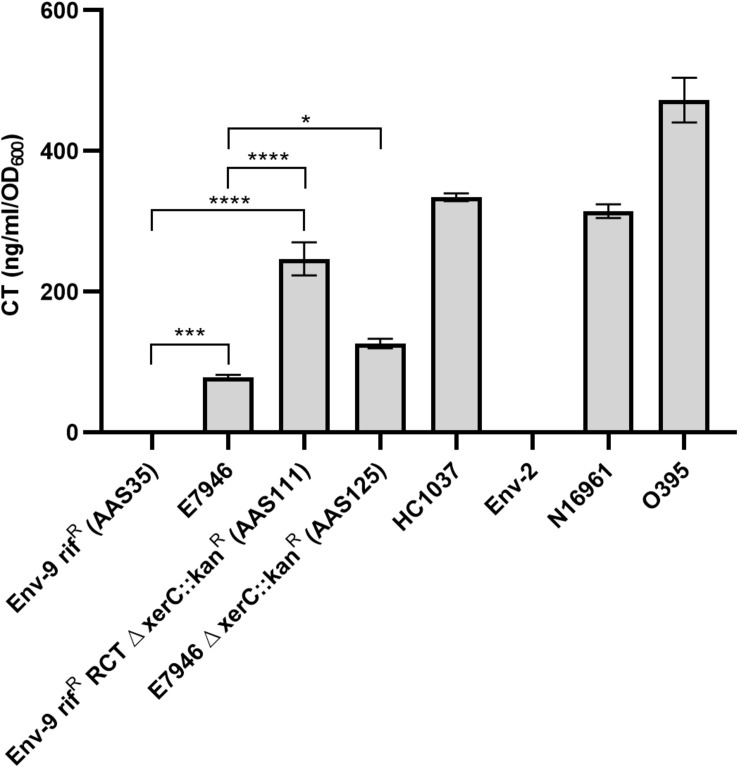
Measurement of cholera toxin (CT) elicited by *Vibrio cholerae* strains in AKI media. GMI- based ELISA method was used to determine CT production by each *V. cholerae* strain. *V. cholerae* strains included in this assay were Env-9 rif^R^ (AAS35), E7946, Env-9 rif^R^ RCT Δ *xerC* (AAS111), E7946 Δ *xerC* (AAS125), a Haitian clinical isolate HC1037, a Haitian non-toxigenic environmental isolate Env-2, N16961, and a classical strain, O395 with the latter two strains were used as positive controls. The results are represented in biological triplicates with mean CT production (ng CT ml^– 1^ OD_600_^– 1^) and are exhibited with standard deviations for each strain. Error bars indicate standard deviation. Asterisks represent statistically significant difference of indicated samples by one-way ANOVA followed by Tukey’s multiple comparison test (^∗^*p* < 0.05, ^∗∗∗^*p* < 0.001, and ^∗∗∗∗^*p* < 0.0001).

To determine if AAS93 (Env-9 rif^R^ RCT Δ *orfU*::*spec*^*R*^) harboring the RCT prophages can produce cholera toxin, we first created an intact CTX prophage in that strain by replacing *spec*^*R*^ with the wild-type *orfU* gene using co-transformation, as described in methods section. This genetic manipulation resulted in the creation of a strain designated as AAS111 (Env-9 rif^R^ RCT Δ x*erC*::*kan*^*R*^) that retained the intact RCT prophages, including the wild-type *orfU* gene. We used an identical technique to generate a similar strain in the background of E7946 resulting in AAS125 (E7946 Δ *xerC*::*kan*^*R*^). Using a standard quantitative cholera toxin (CT) assay, we compared relative CT production among a series of *V. cholerae* strains, including AAS35, E7946, AAS111, AAS125, HC1037, Env-2, N16961, and O395. As shown in Figure 3, AAS111 produced 1.95-fold higher levels of cholera toxin compared to AAS125. Interestingly, AAS125 produced 1.6-fold more cholera toxin compared to its wild-type E7946 strain. As expected a classical *V. cholerae* strain (O395), HC1037 (a Haitian clinical strain) and N16961 produced significantly more CT compared to AAS111. In contrast, wild-type AAS35 and a Haitian non-O1 strain (Env-2) failed to produce CT in identical experimental condition (Figure 3). Our data confirmed that following transformation and acquisition of intact RCT via chitin-induced transformation, AAS111 promoted cholera toxin production that was significantly higher than AAS125.

## Discussion

In this paper, we demonstrated that a non-toxigenic *V. cholerae* O1 strain acquired RCT prophages from a kanR marked donor gDNA via chitin-induced natural transformation rendering that non-toxigenic strain to a toxigenic strain. We also demonstrated that RecA contributed to the homologous recombination facilitating the integration of donor RCT adjacent to *rtxA* gene in the recipient strain. Through this work, we provided an alternative and simpler mechanism by which a non-toxigenic *V. cholerae* O1 strain can acquire RCT, leading to formation of toxigenic strains. Prior studies (Waldor and Mekalanos, 1996; Faruque et al., 2002; Hassan et al., 2010; Faruque and Mekalanos, 2012; Das, 2014) demonstrated that non-toxigenic *V. cholerae* O1 strains acquire RS1ϕ, CTXϕ, and TLCϕ filamentous phages by transduction process. The entire process is complex involving independent and sequential acquisition of each of these phages. These phages use *V. cholerae’s* extracellular appendages, including TcpA, MSHA and TcpA/MSHA as well as helper phages for successful transduction. For example, TcpA is the receptor for CTXϕ infection while TLCϕ uses MSHA as a receptor for successful infection. Moreover, RS1ϕ can use either TcpA or MSHA as receptor for infection. However, while *V. cholerae* O1 expresses TcpA in the human intestine, MSHA is highly repressed in human gut; conversely, in the aquatic environment, MSHA is highly expressed while TcpA is repressed (Tacket et al., 1998; Hsiao et al., 2008). Thus, differential expression of TcpA and MSHA at two distinct ecological niches (human intestine and aquatic environment, respectively) could limit the acquisition of RCT, potentially making it difficult for a non-toxigenic *V. cholerae* O1 strain to convert to a toxigenic strain.

In contrast, our studies demonstrate that a non-toxigenic *V. cholerae* O1 can transform to a toxigenic strain with a single chitin-induced transformation event, particularly in aquatic reservoirs. Intriguingly, previous reports have suggested that heterogeneous biofilm consisting of *V. cholerae* strains could release free gDNA following the natural death and/or predation of biofilm-*V. cholerae* by vibriophages-driven infection (Udden et al., 2008). *V. cholerae* could acquire the free and released gDNA using its natural competency in presence of chitin readily available in aquatic reservoirs (Blokesch and Schoolnik, 2008; Seitz and Blokesch, 2014). Our current study shows that non-toxigenic *V. cholerae* O1 and potentially non-toxigenic non-O1/non-O139 strains can convert to toxigenic strains via chitin-induced natural transformation using exogenous gDNA released by toxigenic *V. cholerae* in the aquatic environment. Indeed, non-toxigenic *V. cholerae* O1/O139 strains are routinely isolated from aquatic reservoirs in both cholera endemic and non-endemic countries. We hypothesize that these non-toxigenic *V. cholerae* O1 strains may convert to toxigenic strain via chitin-induced natural transformation (Mahmud et al., 2014; Zhang et al., 2014; Chowdhury et al., 2015; Azarian et al., 2016). Interestingly, recent reports demonstrated that toxigenic *V. cholerae* non-O1/non-O139 strains, including strains carrying “Haitian” *ctxAB* genes, isolated from clinical and environmental settings caused severe diarrheal disease in India (Bhuyan et al., 2016; Kumar et al., 2018). Emergence of such toxigenic non-O1/non-O139 strains in India may well have resulted via chitin-induced natural transformation using exogenous gDNA from toxigenic *V. cholerae* strains circulating in that country.

The recipient strain AAS35 (Table 1) used in the study is a non-toxigenic *V cholerae* O1 strain with Ogawa serotype. Furthermore, a battery of phenotypic assays distinguishing classical from El Tor biotypes exhibited that this strain retains hybrid phenotypes (some classical phenotypes with additional El Tor phenotypic traits) (Azarian et al., 2016). Despite acquisition of RCT from donor gDNA of AAS56 (Tables 1, 2) using chitin induction, transformant AAS91 (Tables 1, 2) retained phenotypes that is identical to Env-9 as described previously (Azarian et al., 2016). Although Env-9 diverged from its progenitor strain ∼530 years ago (Azarian et al., 2016), it retained ToxR-controlled regulatory genes that regulate, at transcriptional level, the expression of cholera toxin. These genes include *toxR*, *toxS*, and *toxT* (DiRita, 1992; Skorupski and Taylor, 1997). Moreover, Env-9 also retained H-NS protein (Stonehouse et al., 2011) and type II secretion system (Sandkvist et al., 1997) regulating cholera toxin production and secretion.

Remarkably, a *V. cholerae* strain AAS111, a derivative of AAS35 and harboring intact RCT (Table 1 and Figure 3), produced 1.95-fold more cholera toxin compared to its prototype AAS125 strain (genotype, E7946 Δ xerC::kanR) (Table 1 and Figure 3). We currently do not have experimental evidence as to why the same *ctxAB* genes produce significantly different levels of CT in the background of AAS111 compared to AAS125. However, a recent study has demonstrated that a single amino acid change in the ToxT protein significantly affects CT production in *V. cholerae* El Tor strain (Kim et al., 2017). We asked if ToxT or other transcriptional regulatory proteins, including ToxR, ToxS and H-NS protein sustained mutation in AAS111 contributing to increased CT production in that strain relative to AAS 125. We compared protein sequences among AAS35, AAS111, and E7946 (Supplementary Figure S3). We found 100% identity in ToxS and H-NS protein sequences among all three strains examined. In contrast, although we found 100% identity in ToxT protein sequences between AAS35 and AAS111, ToxT protein of E7946 strain has only 81% identity with AAS35 and AAS111 (Supplementary Figure S3). Our observation leads to the hypothesis that such changes in the ToxT protein sequence of AAS111 contributed to the increased production of CT in that strain compared to AAS125. In addition to ToxT, we also observed that the ToxR protein of AAS35 and AAS111 sustained four mutations compared to AAS125 (Supplementary Figure S3). Based on this observation, we cannot rule out the possibility that mutations in the ToxR protein in AAS35 and AAS111 caused increased CT production in that strain. Furthermore, as AAS35 is phenotypically a hybrid of classical and El Tor biotypes, increased CT production by this strain compared to AAS125 could be linked simply to different phenotypic traits of AAS35. Indeed, a previous report suggested that *V. cholerae* hybrid strains produced more CT relative to prototype El Tor variant of *V. cholerae* strains (Ghosh-Banerjee et al., 2010).

There are substantial data supporting the concept that a single toxigenic *V. cholerae* O1 clone (almost certainly introduced by peacekeeping troops from Nepal) was responsible for the massive Haitian cholera epidemic that began in 2010 (Ali et al., 2011; Chin et al., 2011). However, we and others have isolated non-toxigenic O1 and non-O1/O139 strains which are phylogenetically distinct from the epidemic clone (Azarian et al., 2016; Baron et al., 2016). While introduction of toxigenic strains from endemic regions may well serve as the basis for many/most cholera epidemics, the work presented in this paper provides a word of caution, demonstrating that movement of toxin gene into non-toxigenic environmental populations can occur, serving as a driver for emergence of new epidemic strains (Goel et al., 2008). In this context, it is interesting to note that epidemic Haitian *V. cholerae* strains (of presumed Nepalese origin) carry a gene (*ideA*) which limits the ability of these strains to undergo horizontal gene transfer (HGT) via chitin-induced transformation (Dalia, 2018) – which, in turn, may restrict their ability to adapt and evolve under ever-changing environmental conditions. We reason that due to the lack of the *ideA* gene in its chromosome, our ancestral environmental *V. cholerae* strain (Env-9) was able to acquire RCT, rendering itself a toxigenic strain. Epidemic Haitian *V. cholerae* O1 strains also acquired RCT (Table 2), albeit at a ∼100-fold lower frequency, from kan^R^ marked gDNA of E7946 via chitin-induced transformation. We propose that the Env-9 strain and other non-toxigenic *V. cholerae* O1 and/or non-O1 strains persisting in Haiti aquatic reservoirs could become toxigenic following the acquisition of intact RCT released from circulating toxigenic Haiti *V. cholerae* strains via chitin-induced transformation. Under such a scenario, there is the potential for generation of a new wave of toxigenic/progenitor strains, which, in turn, might drive a new wave of cholera in Haiti.

## Data Availability Statement

The datasets generated for this study can be found in the GenBank accession nos. CP042299 and CP042300.

## Author Contributions

AA conceived, designed, and overall supervised the research work, and wrote the manuscript. SS-R, MA, and SB designed and conducted the experiments. AA, SS-R, MA, and SB analyzed and interpreted the research results, including statistical analysis. AA, SS-R, MA, SB, and JM critically revised, edited, and approved the manuscript.

## Conflict of Interest

The authors declare that the research was conducted in the absence of any commercial or financial relationships that could be construed as a potential conflict of interest.
