# Supplementary material for: Conversion of a recA-Mediated Non-toxigenic Vibrio cholerae O1 Strain to a Toxigenic Strain Using Chitin-Induced Transformation
Source: Front Microbiol. 2019 Nov 7;10:2562. doi: 10.3389/fmicb.2019.02562 (PMC6854035; doi:10.3389/fmicb.2019.02562)
Supplement: Supplementary file 1 [file Data_Sheet_1.docx]

Supplementary Material

# Supplementary Figures and Tables


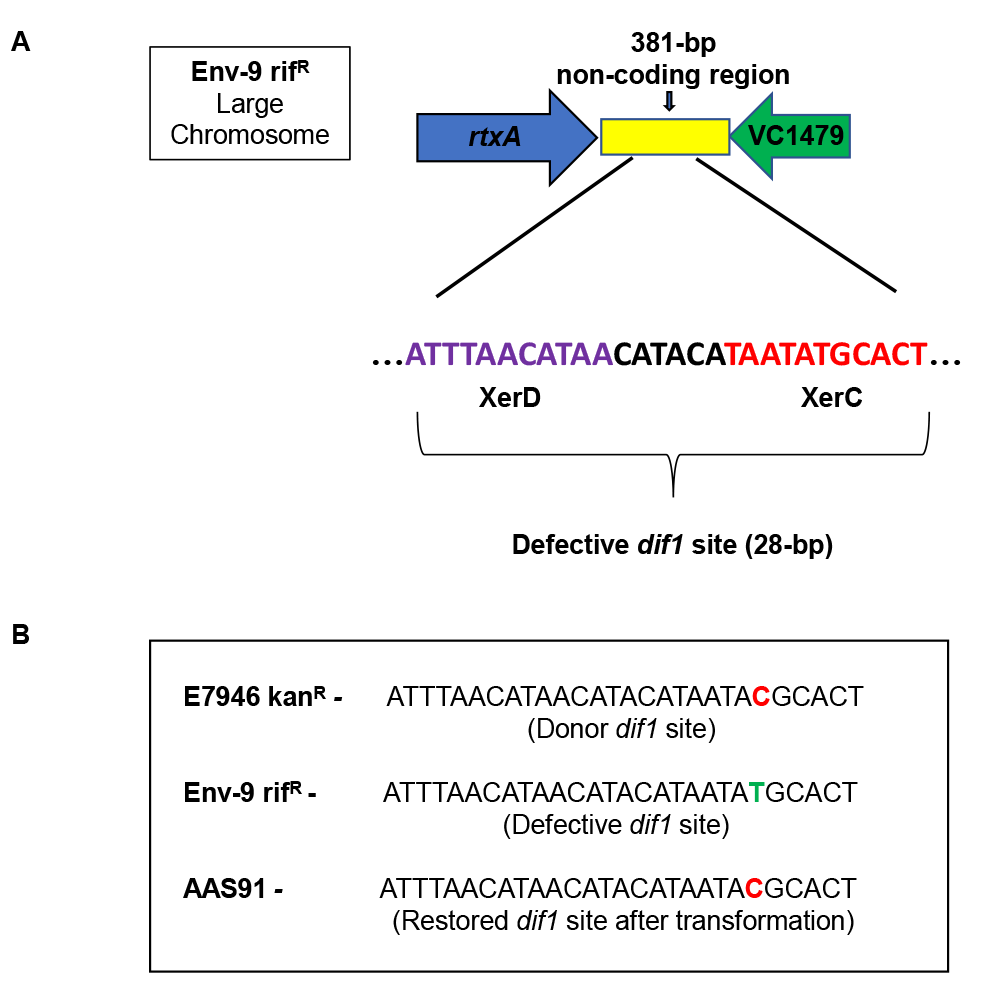
**1.1 Supplementary Figures**

**Supplementary Figure 1.**

Conversion of defective *dif1* site present in the large chromosome of wild-type Env-9 rif^R^ (AAS35, **Table 1**) into a restored *dif1* site following chitin-induced transformation. **(A)** Env-9 rif^R^ strain (AAS35) lacking RCT prophages retained a 381-bp non-coding region between ORFs VC1451 (*rtxA*) and VC1479 encoding a hypothetical protein. Of 381-bp, 28-bp (shown in the figure) harbors defective *dif1* site with sequences required for the binding of XerC and XerD recombinases. **(B)** Box displaying comparative *dif1* sequences between E7946 kan^R^ (AAS56) [source of donor gDNA strain], recipient Env-9 rif^R^ strain (AAS35) and a chitin-induced transformant (AAS91).


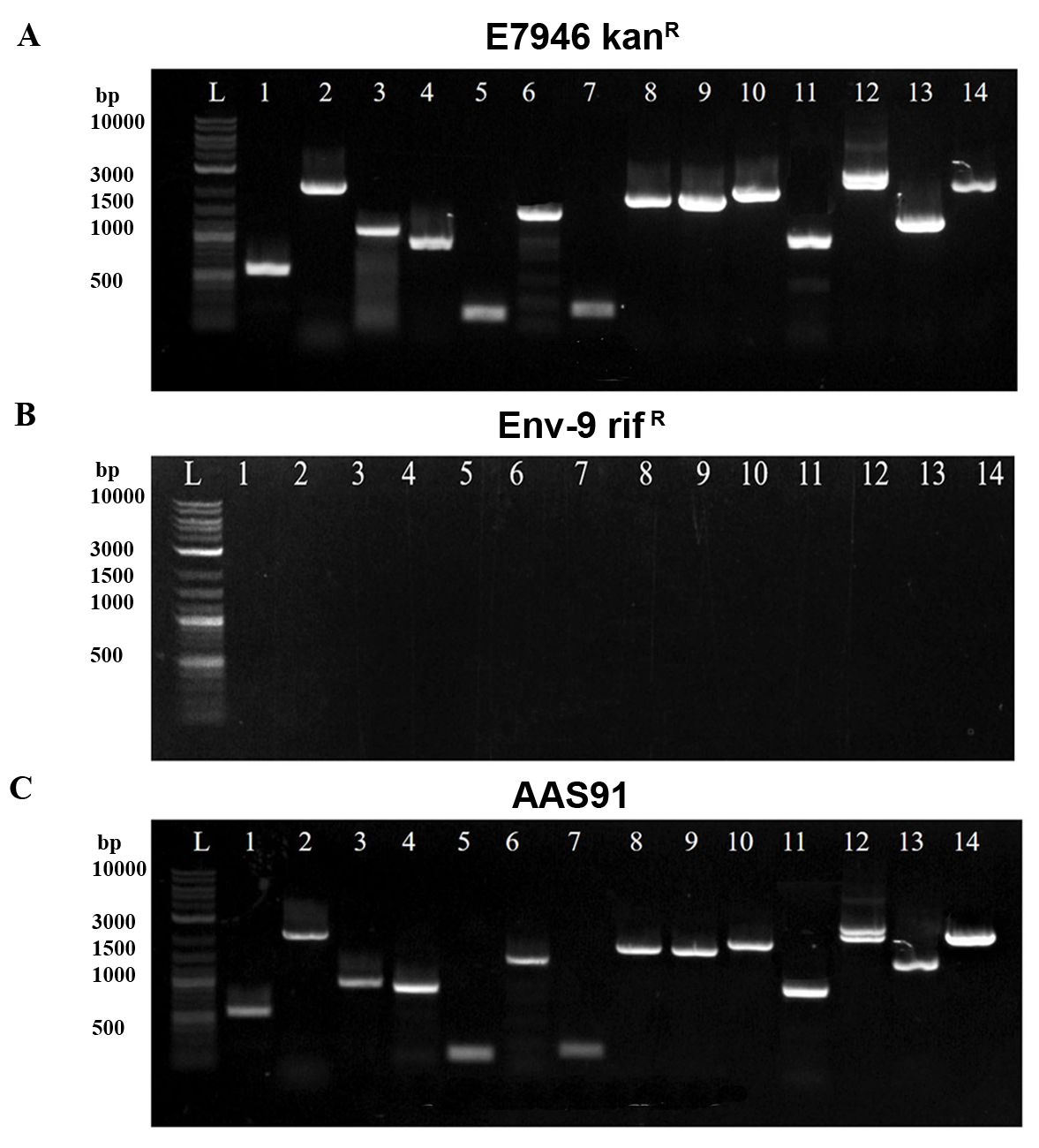


**Supplementary Figure 2.**

Detection of all genes encompassing RCT prophages using PCR. We designed fourteen convergent PCR primer sets to amplify 14 PCR products spanning *V. cholerae*’s genomic segment containing 27 genes located between *rtxA* gene and VC1479. Images of agarose gel indicating all 14 PCR products: **(A)** E7946 kan^R^ (AAS56, donor gDNA), **(B)** Env-9 rif^R^ (AAS35, recipient strain), and **(C)** AAS91 (transformant resulted from chitin-induced transformation). Lanes: L, 1 kb DNA ladder; 1, *rtxA-rstC*; 2, *rstB1-rstR*; 3, *ctxB-ctxA*; 4, *zot*; 5, *ace*; 6, *orfU*; 7, *cep*; 8, *rstA2-rstR*; 9, VC1465-VC1468; 10, *cri-tlcR*; 11, intergenic region of *tlcR* and VC1471; 12, VC1471- VC1474; 13, *cri-tlcR*; and 14, *orfA*-VC1479

**A**


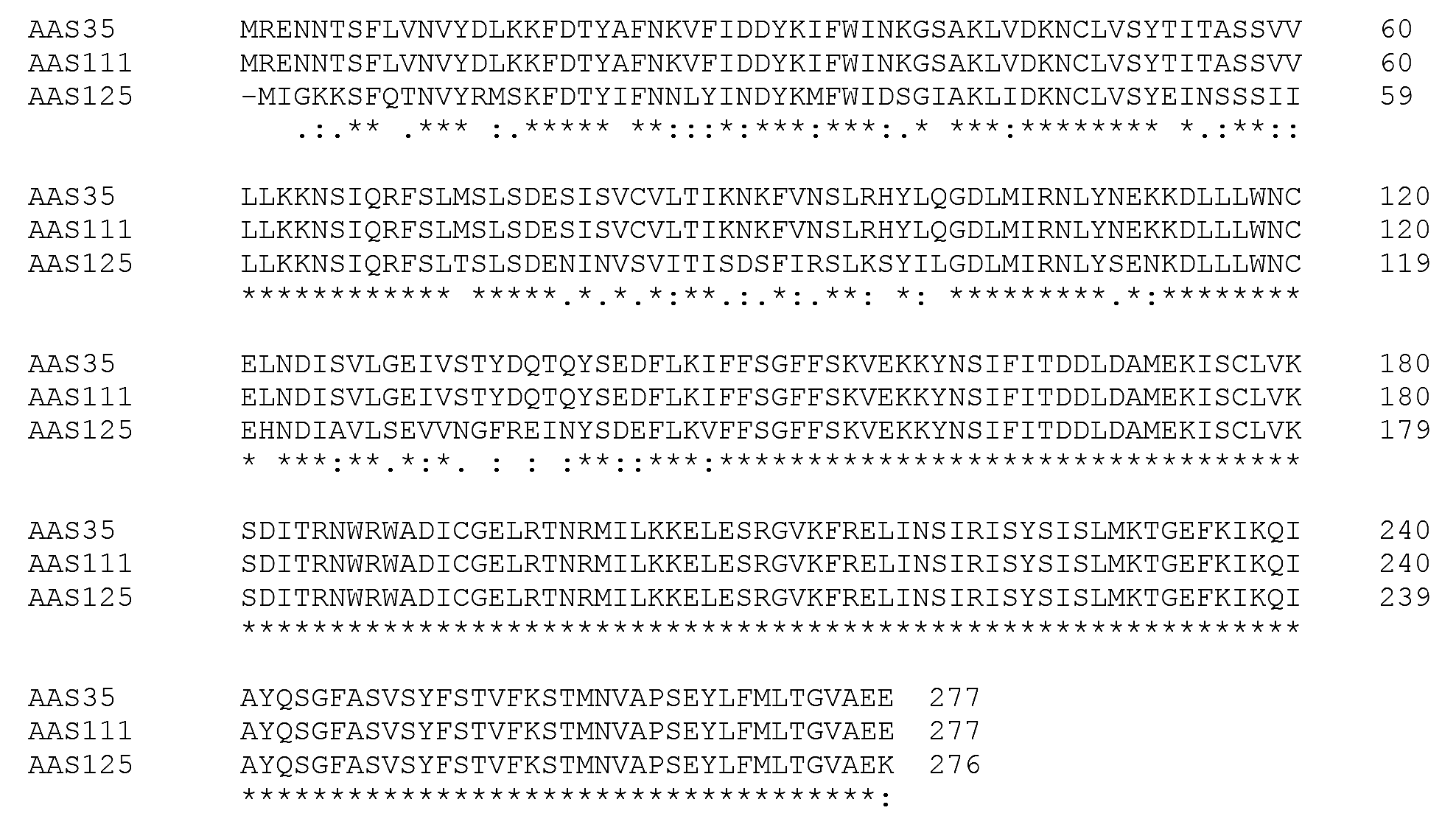


**B**


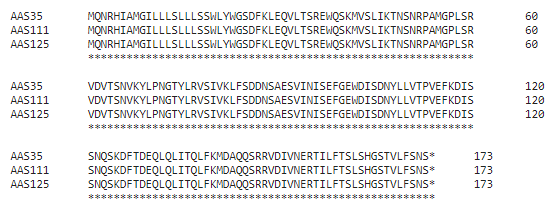


**C**


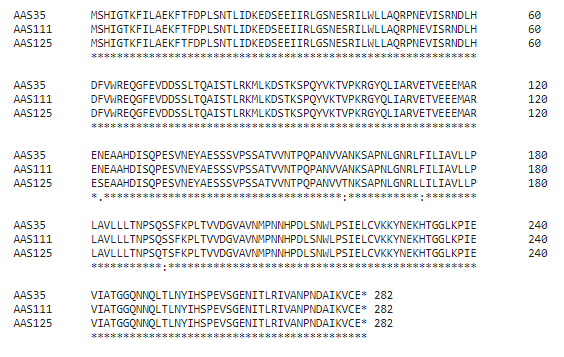


**D**


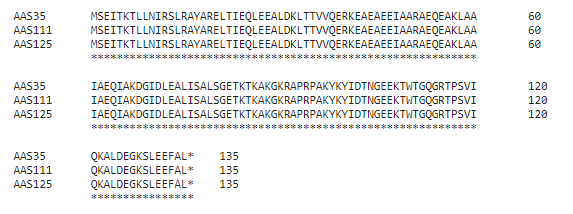


**Supplementary Figure 3.**

Comparison of protein sequences among AAS35 (Env-9 rif^R^), AAS111 (Env-9 rif^R^ RCT Δ *xerC*::*kan^R^*) and AAS 125 (E7946 Δ *xerC*::*kan^R^*). We used Clustal Omega to compare the protein sequences. (**A**) ToxT (**B**) ToxS (**C**) ToxR and (**D**) H-NS. Symbols numbers in the right margin represent the positions of the corresponding amino acid residues in the full-length protein. *, **:** and . indicate identical, similar and less similar amino acids, respectively.

**1.2 Supplementary Table**

**Supplementary Table 1**: PCR primers used in this study.

| Primer^a,b^ | Description | | Sequence (5’→ 3’) |
| --- | --- | --- | --- |
| Primers used for making mutants by SOE PCR | | | |
| aali 563 | *Kan^R^ / Spec^R^-F* | | ATTCCGGGGATCCGTCGAC |
| aali 564 | *Kan^R^ / Spec^R^-R* | | TGTAGGCTGGAGCTGCTTC |
| aali 651 | *orfU-*F1 | | ACTAATTGCGGCAATCGCATG |
| aali 655 | *orfU-*R1 | | **GTCGACGGATCCCCGGAAT**CATTCTGGCCGCCATGCTCAT |
| aali 656 | *orfU-*F2 | | **GAAGCAGCTCCAGCCTACA**CAAAGAGACCCAGCCACAACT |
| aali 654 | *orfU-*R2 | | CGCATTAAGGCGGTATGTCAT |
| aali 551 | *xerC-*F1 | | CTGAGCAAGTTCCACTCGCCGATT |
| aali 552 | *xerC-*R1 | | **GTCGACGGATCCCCGGAAT**CTGGGTATACAGGCTCAACCCTTT |
| aali 553 | *xerC-*F2 | | **GAAGCAGCTCCAGCCTACA**GTGTATGACCAAGCGCATCCT |
| aali 554 | *xerC-*R2 | | CGGGCGATGACGCATCATATT |
| aali 555 | *xerD-*F1 | | TGAGAGAGGGCGTTGTTTTCAATC |
| aali 556 | *xerD-*R1 | | **GTCGACGGATCCCCGGAAT**CTTGGGCATAGTGATTTATCGACC |
| aali 557 | *xerD-*F2 | | **GAAGCAGCTCCAGCCTACA**CTCCTGCAAACCAGCAAAGCT |
| aali 558 | *xerD-*R2 | | AGGCTTGATGCCTCCTGTTAACTG |
| aali 732 | *recA-*F1 | | CTTTGGTCGAGTGGCGTCAA |
| aali 733 | *recA-*R1 | | **GTCGACGGATCCCCGGAAT**GGCCGCAAGTGCCTTCTGTTTA |
| aali 734 | *recA-*F2 | | **GAAGCAGCTCCAGCCTACAC**AACTGACTGGTGAAACGGC |
| aali 735 | *recA-*R2 | | CCATCCAGCACTTGGCCTGATA |
| Primers used for identifying all the genes present in the CTX composite genetic element | | | |
| aali 537 | 1F | | GGATTGATGGTGTAATCGCGT |
| aali 538 | 1R | | TACGATAGCCTAGAAGAC |
| aali 812 | 2F | | GCTCAGTCAATGCCTTGAGTTG |
| aali 197 | 2R | | GCACCATGATTTAAGATGCTC |
| aali 078 | 3F | | CTCAGACGGGATTTGTTAGGCACG |
| aali 045 | 3R | | TTGCTTCTCATCATCGAACC |
| aali 541 | 4F | | TCGCTTAACGATGGCGCGTTTT |
| aali 542 | 4R | | AACCCCGTTTCACTTCTACCCA |
| aali 543 | 5F | | TGGCTTGTGATCAAGCTCG |
| aali 544 | 5R | | CATCAAAGCCTGAAGCACG |
| aali 657 | 6F | | GCCACGTAAAGCCATCA |
| aali 658 | 6R | | GATACGGTGATTGCGCTTGG |
| aali 547 | 7F | | GCTACATGTTTAGCTCACTG |
| aali 548 | 7R | | TTTAGCCTTACGAATTAAGCC |
| aali 550 | 8F | | GGTTGAGGTTTATCCAATGG |
| aali 197 | 8R | | GCACCATGATTTAAGATGCTC |
| aali 611 | 9F | | CTCTAGGTTTAGTACGGTGGC |
| aali 583 | 9R | | GCATTAGCCATCGCAAAG |
| aali 585 | 10F | | CTCTTGGTTGATTGGCCGCTT |
| aali 587 | 10R | | GAGAGCTTCTGGAGAGAG |
| aali 818 | 11F | | CATAAGCCGCTATTAGAG |
| aali 466 | 11R | | CAGGGCAATTTGTCGGT |
| aali 539 | 12F | | ACATAATGCGCACTAGGA |
| aali 584 | 12R | | GACTCGTATTGACTGCACG |
| aali 531 | 13F | | GCCGACATCCAACAACTC |
| aali 532 | 13R | | GTCGTCTTGTACGTTTTGTAC |
| aali 528 | 14F | | GTACAAAACGTACAAGACGAC |
| aali 571 | 14R | | CACAAGTTCAAACTCGCC |
| Primers used for cloning | | | |
| aali 1053 | | *recA*-F | CGC*CTCGAG*ATGGACGAGAATAAACAGAAG |
| aali 1054 | | *recA*-R | CGC*GAATTC*TTAAAACTCTTCTGGCACCG |
| Primers used for sequencing | | | |
| aali 1057 | | pBAD-F | ATGCCATAGCATTTTTATCC |
| aali 1058 | | pBAD-R | GATTTAATCTGTATCAGG |

^a^Regions overlapped with the kan^R^ / spec^R^ cassette are indicated in bold.

^b^Restriction endonuclease sites incorporated in the primer sequence are marked in italics.

**Supplementary Table 2.** Transformation frequency of distinct mutants (recipient strains) genetically derived from *V. cholerae* Env-9 rif^R^ (AAS35) strain with genomic DNA (gDNA) obtained from a donor *V. cholerae* E7946 Δ *orfU*::*spec^R^* strain (AAS72) via chitin induction. Data represent multiple independent transformation experiments. Complementation of Env-9 rif^R^ Δ *recA*::*kan^R^* (AAS74b) with a wild-type *recA* gene cloned into pBAD vector (pSMA4) restoring the acquisition of RCT in that mutant

| Donor gDNA | Recipient strain | Range of transformation frequency |
| --- | --- | --- |
| E7946 Δ *orfU*::*spec^R^* | Env-9 rif^R^ Δ *xerC* ::*kan*^R^ | 1.44 x 10^-5^ - 4.16 x 10^-6^ |
| E7946 Δ *orfU*::*spec^R^* | Env-9 rif^R^ Δ *xerD* ::*kan*^R^ | 1.62 x 10^-5^ - 3.33 x 10^-6^ |
| E7946 Δ *orfU*::*spec^R^* | Env-9 rif^R^ Δ *recA* ::*kan*^R^ | 0 |
| E7946 Δ *orfU*::*spec^R^* | Env-9 rif^R^ Δ *recA* ::*kan*^R^ (pBAD) | 0 |
| E7946 Δ *orfU*::*spec^R^* | Env-9 rif^R^ Δ *recA* ::*kan*^R^ (pSMA4) | 7.10 x 10^-5^ - 2.16 x 10^-6^ |
